# Evaluation of the Applicability of Synthetic Data in the Development of Colorectal Cancer Survival Prediction Models: External Validation of Advanced Machine Learning Models Based on National Cancer Data Center Data

**DOI:** 10.2196/86087

**Published:** 2026-07-07

**Authors:** Yujeong Jang, Jae Hoon Kwon, Heeyong Kim, You-Jin Joung, Junho ‍Nang, Chang Hyun Kim

**Affiliations:** 1 Department of Regulatory Science, Kyung Hee University, Seoul, Republic of Korea; 2 Department of Surgery, Chonnam National University Hwasun Hospital, 322 Seoyang-ro, Hwasun, 58128, Republic of Korea; 3 C&R Research, Seoul, Republic of Korea; 4 Clinical Trial Center, Institute for Biomedical Science, Chonnam National University Hwasun Hospital, 322, Seoyang-ro, Hwasun-eup, Hwasun-gun, Jeollanam-do, Republic of Korea, 82 10-8940-1365

**Keywords:** synthetic data, domain adaptation, colorectal cancer, machine learning, extreme gradient boosting, light gradient boosting machine, survival prediction model, external validation

## Abstract

**Background:**

Limited data availability and privacy constraints hinder the development of robust survival prediction models for personalized treatment. Synthetic data offers a promising solution, preserving the statistical properties of real clinical data.

**Objective:**

This study aimed to quantitatively assess the feasibility of using synthetic data for survival prediction by evaluating model transfer performance to real-world hospital data, with a focus on model transfer strategies.

**Methods:**

We developed and validated colorectal cancer survival prediction models using the National Cancer Data Center (NCDC) synthetic data (30,683 patients from 3 Korean institutions) for pretraining and real hospital data (2170 patients from Hwasun Jeonnam University Hospital) for external validation. We evaluated 3 model transfer strategies—domain adaptation, zero-shot, and ensemble—using extreme gradient boosting (XGBoost) and light gradient boosting machine (LightGBM). In total, 48 model configurations were tested, defined by the combination of algorithms (LightGBM and XGBoost), sampling technique (no-sampling, random undersampling [RUS], and synthetic minority oversampling technique combined with edited nearest neighbors [SMOTEENN]), model type (baseline, domain adaptation, zero-shot, and ensemble), and optimization objective (area under the precision-recall curve [AUPRC] and *F*_1_). The outcome was 7-year overall survival, evaluated using the AUPRC and Brier scores. Performance was compared against a hospital-only baseline using absolute values and deltas (ΔAUPRC and ΔBrier). Differences and corresponding 95% CIs were estimated on the held-out test set using 2000 bootstrap samples.

**Results:**

Zero-shot application reduced the AUPRC in most settings, and any marginal improvements observed in the remaining settings were not statistically significant. In contrast, the domain adaptation model improved AUPRC in 8/12 combinations, with 4 statistically significant gains; the best setting (XGBoost+RUS+*F*_1_ optimization) achieved AUPRC=0.5391 (Δ+0.1474; *P*<.001). The soft ensemble increased AUPRC in 7/12 combinations, with 3 statistically significant gains; the best setting (XGBoost+RUS+AUPRC optimization) achieved AUPRC=0.5060 (*Δ*+0.1258, *P*=.002). For calibration, Brier scores improved in most domain adaptation and ensemble combinations, with a substantial proportion reaching statistical significance.

**Conclusions:**

When domain adaptation using local hospital data was applied, the model pretrained on synthetic data exhibited similar performance to the hospital-only baseline across various settings. This study demonstrates the methodological utility of a model transfer approach using NCDC synthetic data in a setting with limited data sharing. At the same time, it clarifies that while synthetic data can serve as a complement to local clinical data, it is not a substitute for real-world clinical models.

## Introduction

Advances in genomics and AI enable the development of personalized therapeutic strategies that integrate patients’ unique genetic information, physiological characteristics, and environmental factors [1]. Personalized therapy is advancing the precision medicine paradigm, highlighting the growing importance of predictive models for individualized treatment [2]. Reliable predictive models for patient-centered personalized treatment require access to sufficient patient data with various clinical characteristics [3]. In addition, evaluating these models through external verification is essential for their application in clinical settings [4]. However, accessing clinical data from real-world health care environments poses significant challenges. Clinical data, including patient disease information and medical records, are subject to privacy-related legal restrictions that limit their acquisition [5]. Additionally, data from a single health care organization have limited generalizability, and sharing clinical data across institutions presents technical and institutional challenges [6]. These constraints hinder model training, performance evaluation, and external validation, limiting the development of patient-specific models. Previous survival prediction model studies typically used patient data collected from medical institutions [7]. Although this approach is valuable for clinical applications owing to its direct reflection of real conditions and disease progression of patients, it is limited by insufficient external validation, as most studies rely solely on internal validation [8].

The use of synthetic data has been proposed as a potential solution to these challenges. Synthetic data retain the variable characteristics and statistical distributions of real-world patient data while being truly nonidentifiable because the data are unrelated to any real individual [9,10]. Therefore, they are free from legal and ethical privacy constraints and can be used to generate large-scale data encompassing diverse patient population characteristics, addressing data sparsity [11]. Synthetic data can be used to proactively model and validate various medical situations that are difficult to simulate in clinical environments [12]. In addition, by applying large-scale synthetic data to the training and validation processes of survival prediction models, the model generalization performance can be improved [13]. Recent studies have reported that synthetic data can reproduce statistical properties similar to real clinical data [14,15]. Specifically, current research on synthetic data focuses on medical image or data augmentation methods that generate synthetic data from real data and combine it with the original data for training [16]. However, there are limited studies that developed predictive models using synthetic data and validated them on real medical data to evaluate the versatility and generalizability [17]. Furthermore, when external data that were not part of synthetic data generation are used for validation, domain shifts may occur owing to differences in hospital-specific patient characteristics, treatment patterns, and other factors [18]. To address the distributional differences between synthetic and real-world hospital data, we applied a domain adaptation approach. Domain adaptation is an umbrella term for a category of transfer learning strategies to mitigate performance degradation due to domain shifts [19]. In this study, we used domain adaptation as a method for transferring knowledge learned from synthetic data to real clinical data.

In this context, this study aimed to evaluate the clinical applicability of predictive models based on synthetic data. The effectiveness of applying a model pretrained on synthetic data to actual hospital data was quantitatively evaluated. Specifically, we focused on providing empirical evidence for the clinical utility of synthetic data and identifying the conditions and limitations of its application in real health care settings.

## Methods

### Data Source and Study Population

We used 2 types of data sources for this study. Synthetic data were based on the “Colorectal Cancer Clinical Library Artificial Dataset” (the synthesized data is archived on the National Cancer Data Center [NCDC] portal) [20]. This dataset is a high-quality synthetic resource that replicates the structure and statistical characteristics of real-world medical data. It was developed based on real-world patient information collected from 3 leading Korean medical institutions specializing in cancer (Samsung Medical Center, Yonsei University Severance Hospital, and National Cancer Center) using the real-world time-series generative adversarial network (RTSGAN) technology [21]. Hospital data comprised clinical information of patients who underwent colorectal cancer surgery at the Hwasun Jeonnam University Hospital in South Korea. This dataset is derived from real patient treatment experiences and medical records, containing detailed clinical indicators and postoperative follow-up results. Study participants included in the synthetic and hospital data were selected based on the following inclusion and exclusion criteria. Inclusion criteria were as follows: (1) patients diagnosed with colorectal cancer between 2009 and 2014, and (2) patients undergoing surgery. The exclusion criterion was patients with missing survival information.

### Outcome Definition and Input Variables

The primary outcome of this study was 7-year overall survival data. The synthetic dataset included 31 variables, while the hospital dataset contained 48 variables. In total, 12 variables common to both datasets were selected as the input variables for model training. The baseline model was intentionally restricted to 12 shared variables common to both datasets to allow for a methodologically controlled comparison between the hospital and synthetic datasets. This feature-constrained design is intended to minimize confounding factors that may arise due to differences in variable availability and information content [22,23]. The final 12 variables were sex, age, stage (0, I, II, III, or IV), adenocarcinoma histology, vascular invasion, lymph node invasion, perineural invasion, tumor location (colon, junction, or rectum), preoperative carcinoembryonic antigen (log-transformed), postoperative chemotherapy (FOLFOX, FOLFIRI, GELOX, or other), postoperative radiation therapy, and number of resected metastatic lymph nodes.

### Data Processing

All categorical variables were encoded as one-hot. Age was used as a binary indicator by interval (<20, 20‐29, …, 80‐89, ≥90). Input variables or outcomes with missing values were excluded from the analysis, based on the principles of complete case analysis.

### Domain Adaptation

To minimize the domain gap between synthetic and hospital data, we applied a domain adaptation technique, a form of transfer learning. Domain adaptation broadly refers to solving the problem of performance degradation caused by the difference in distribution between the source and target domains. In this study, we implemented domain adaptation through parameter transfer. It was first trained on synthetic data (source domain) and then fine-tuned on hospital data (target domain) using a low learning rate and regularization. This approach allows the model to adapt to hospital-specific characteristics using limited real-world data while retaining generalizable patterns learned from large-scale synthetic data. In this study, we did not apply feature-level alignment or adversarial domain adaptation techniques. Instead, we focused on parameter-level adaptation, which is particularly well-suited to tabular clinical data, where explicit feature alignment across domains is difficult, and privacy constraints may limit access to joint feature distributions [24-26]. Furthermore, to determine the presence of a domain gap, we performed the Kolmogorov-Smirnov test for continuous variables and chi-square (or Fisher exact test, where expected cell counts were less than 5) independence tests for categorical variables.

### Data Splitting

**Figure 1.**
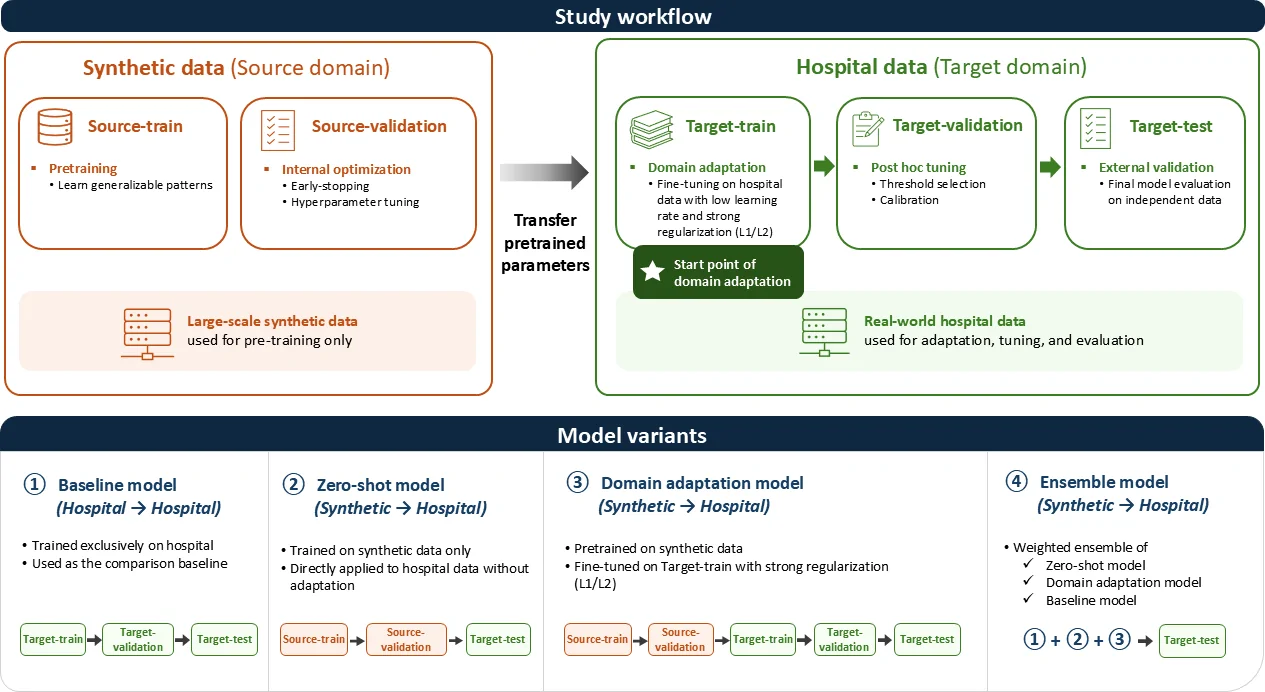
Data splitting procedure and domain adaptation procedure.

In this study, synthetic data were used for pretraining and internal optimization, while hospital data were used for domain adaptation, threshold selection, probability calibration, and independent final evaluation. Therefore, the synthetic data were split into source-train or source-validation data based on the year of diagnosis, and the hospital data were sorted by the year of diagnosis and split into target-train, target-validation, or target-test using a 6:2:2 3-partition (Figure 1).

Synthetic data were split into source-train and source-validation sets based on the year of diagnosis and were used for pretraining and internal optimization. Hospital data were chronologically divided into target-train, target-validation, and target-test sets using a 6:2:2 ratio and were used for domain adaptation, threshold selection, probability calibration, and independent final evaluation. The pretrained model was fine-tuned on target-train data through domain adaptation, with threshold selection and calibration on target-validation, and independent final evaluation on the held-out target-test set. Four model variants were evaluated: a baseline model (Hospital → Hospital), a zero-shot model (Synthetic → Hospital), a domain adaptation model (Synthetic → Hospital), and an ensemble model combining predictions from multiple models.

### Handling Class Imbalance

According to the National Cancer Information Center, the 5-year relative survival rate for colorectal cancer is 75% [27]; however, the outcome measured in this study showed similarly high survival expected at 7 years. To address this class imbalance, sampling techniques—including no sampling, random undersampling (RUS) [28], and the synthetic minority oversampling technique combined with edited nearest neighbors (SMOTEENN) [29]—were applied to the source and target training datasets. To preserve the true prevalence during evaluation, no sampling technique was applied to the validation and test datasets [30].

### Algorithms

We used light gradient boosting machine (LightGBM) and extreme gradient boosting (XGBoost). Specifically, LightGBM offers fast learning, effective handling of imbalanced data through is_unbalance/weight, and supports efficient stopping and fine-tuning. XGBoost reliably controls overfitting and imbalanced data with L1/L2 normalization and scale_pos_weight, and is suitable for normalization fine-tuning with the xgb_model load. Furthermore, it has a high training speed to compare various combinations quickly [31,32]. Considering our settings, moderate class imbalance, domain shift, and tabular features, these 2 algorithms are natural choices.

For domain adaptation, fine-tuning was performed by first using a model trained on synthetic data as an initial model and then training additional trees using hospital data. In this process, the previously trained tree structure was kept fixed without modification, and the adaptation to the hospital data was done by adding new trees. In order to preserve the knowledge learned from the synthetic data as much as possible and prevent overfitting due to domain shift, a low learning rate and strong L1/L2 normalization were applied in the fine-tuning phase.

### Model Variants

We configured four model variants as follows:

1. Baseline model (Hospital→Hospital): It served as the reference standard and was trained and evaluated exclusively on hospital data.
2. Domain adaptation model (Synthetic→Hospital): This is the main model of this study, and it uses pretrained weights from synthetic data as initialization and applies domain adaptation with fine-tuning on hospital data using low learning rates and strong regularization (L1/L2).
3. Comparator models:
  - Zero-shot model (Synthetic→Hospital): This model is trained on synthetic data and directly applied to hospital data without any adaptation.
  - Ensemble model (Synthetic→Hospital): This model combines 3 components through weighted ensemble—a pretrained model from synthetic data, a fine-tuned model on hospital data, and a model trained from scratch on hospital data.

### Optimization Objective

Each model was optimized for 2 objectives—area under the precision-recall curve (AUPRC) and *F*_1_-scores. On the target validation set, hyperparameter tuning, early stopping, and threshold selection were performed by maximizing each metric.

### Model Evaluation

In this study, 48 combinations were tested as follows: algorithm (LightGBM, XGBoost)×sampling technique (no-sampling, RUS, SMOTEENN)×model (baseline, domain adaptation, zero-shot, ensemble)×optimization objective (AUPRC, *F*_1_).

The performances of the domain adaptation and baseline models were compared in the primary analysis. To objectively evaluate the domain adaptation effect, zero-shot and ensemble models were included as comparators. The main evaluation metrics are the AUPRC and the Brier score; for each experimental combination, we determined absolute values on the test set and changes relative to the baseline (ΔAUPRC and ΔBrier score). The Δ values presented in this study are not the arithmetic differences between point estimates. They represent the mean difference in performance between models, estimated by bootstrapping the test set 2000 times. This approach was adopted to provide more robust estimates of performance differences. The 95% CIs were derived from the 2.5th and 97.5th percentiles of the bootstrap distribution, and 2-sided *P* values were obtained as twice the minimum of the proportion of bootstrap resamples with a difference of zero or less and the proportion with a difference of zero or greater. The AUPRC was chosen to measure discriminative performance in the presence of class imbalance. In situations of class imbalance, the precision-recall approach further highlights the limitations of receiver operating characteristic–based evaluation [33]. The Brier score was included to assess whether the predicted probability matches the observed outcome. Models with similar discriminative power can have significant differences in probability accuracy and real-world interpretability; therefore, a comprehensive evaluation of clinical prediction models should consider both discriminative power and degree of calibration [34]. The AUROC, precision, recall, *F*_1_, *F*_2_, accuracy, Matthews correlation coefficient, and specificity were reported as secondary metrics.

### Software and Statistical Analysis

Data preprocessing and extraction were performed using SAS (version 9.4; SAS Institute), while all subsequent analyses were conducted in Python (version 3.8.10; Python Software Foundation) within a controlled computational environment ensuring reproducibility. All analyses in this study were performed in a controlled computational environment with fixed software versions, an object-oriented pipeline structure, and systematic logging to ensure reproducible experiments for multiple model combinations [35].

Data management:

- SAS 9.4: Used for initial data extraction, cleaning, and preprocessing of hospital clinical data. DATA manipulation and PROC procedures were performed using DATA steps and quality control SQL.

Python environment:

- Machine Learning: scikit-learn (1.2.0), XGBoost (1.7.3), and LightGBM (3.3.5)
- Data Processing: pandas (1.5.3), numpy (1.24.2)
- Statistical Analysis: scipy (1.10.1), statsmodels (0.13.5)
- Sampling Techniques: imbalanced-learn (0.10.1)
- Visualization: matplotlib (3.6.3), seaborn (0.12.2)
- Performance Metrics: scikit-learn, custom implementation for medical-specific metrics

### Ethical Considerations

This retrospective study was approved by the Institutional Review Board of Chonnam National University Hwasun Hospital (IRB file CNUHH-2024‐113). The ethics committee of Chonnam National University Hwasun Hospital waived the requirement for informed consent because the study involved a retrospective review of anonymized medical data. All data used in the analysis were fully deidentified before access by the research team, and no personally identifiable information was available. Data were stored on secure, access-restricted systems to ensure privacy and confidentiality. No financial or other compensation was provided to participants, as this study involved secondary analysis of existing clinical data. The manuscript and all supplementary materials do not contain any images or information that could lead to the identification of individual participants.

## Results

### Cohort

**Figure 2.**
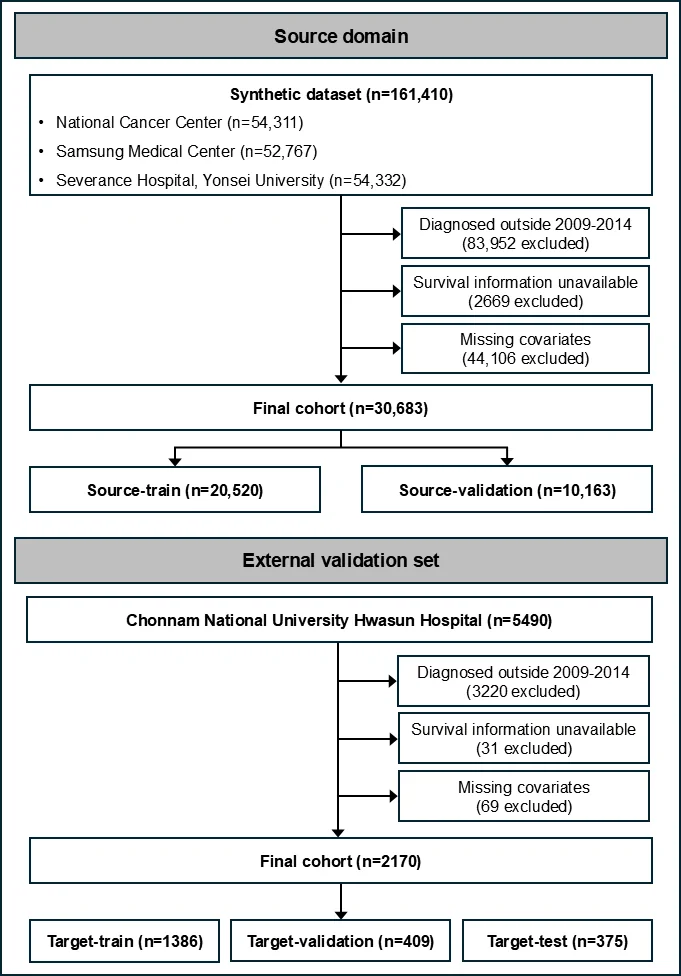
Flowchart of participant selection.

In this study, synthetic data comprising 161,410 records—54,311 from the National Cancer Center, 52,767 from Samsung Medical Center, and 54,332 from Yonsei University Medical Center—were used for pretraining. During preprocessing, 44,106 records were excluded due to missing values in essential covariates. After applying selection and exclusion criteria and data preprocessing, the final dataset for survival model prediction included 30,683 patients. The external validation dataset from the Hwasun Jeonnam National University Hospital included 5490 surgical patient records, of which 2170 were used in the analysis. A flowchart of the participant selection process is illustrated in Figure 2.

### Domain Shift Assessment

Distribution comparison of the 2 datasets (Table 1) showed statistically significant differences for all variables (*P*<.05). For patient characteristics, the hospital data showed higher age (median 67, IQR 59-74 y vs median 63, IQR 55-70 y; *P*<.001) and a slight but statistically significant difference in sex (*P*=.04). The prevalence of adenocarcinoma (histology) was significantly higher in the hospital data than in the synthetic data (97.2% vs 55.6%, *P*<.001), and perineural invasion (29.4% vs 0.7%, *P*<.001) and vascular invasion (19.2% vs 9.4%, *P*<.001) were higher in hospital data. Stage distribution differed significantly (*P*<.001), with a higher proportion of stage III patients in the hospital cohort, which comprised predominantly high-risk patients. A clear domain shift was observed between synthetic and hospital data, necessitating the use of domain adaptation techniques to ensure model performance in real-world settings.

**Table 1.** Demographic and clinical characteristics of patients with colorectal cancer using synthetic and hospital-based data.

Variables	Synthetic data (n=30,683)	Hospital data (n=2170)	*P* value^a^
7-year overall survival, n (%)			<.001
No	1943 (6.3)	431 (19.9)	
Yes	28,740 (93.7)	1739 (80.1)	
Sex, n (%)			.04
Male	18,279 (59.6)	1341 (61.8)	
Female	12,404 (40.4)	829 (38.2)	
Age (y), median (IQR)	63 (55‐70)	67 (59‐74)	<.001
Stage, n (%)			<.001
0	587 (1.9)	82 (3.8)	
I	9928 (32.4)	530 (24.4)	
II	10,599 (34.5)	747 (34.4)	
III	6995 (22.8)	789 (36.4)	
IV	2574 (8.4)	22 (1.0)	
Adenocarcinoma (histology), n (%)			<.001
Non-adenocarcinoma	13,627 (44.4)	60 (2.8)	
Adenocarcinoma	17,056 (55.6)	2110 (97.2)	
Vascular invasion, n (%)			<.001
No	27,791 (90.6)	1753 (80.8)	
Yes	2892 (9.4)	417 (19.2)	
Lymph node invasion, n (%)			<.001
No	22,501 (73.3)	1742 (82.3)	
Yes	8182 (26.7)	428 (19.7)	
Perineural invasion, n (%)			<.001
No	30,474 (99.3)	1531 (70.6)	
Yes	209 (0.7)	639 (29.4)	
Tumor location, n (%)			<.001
Colon	18,777 (61.2)	1404 (64.7)	
Rectum	8869 (28.9)	681 (31.4)	
Junction	3037 (9.9)	106 (4.9)	
Number of resected metastatic lymph nodes, median (IQR)	0.0 (0.0‐0.0)	0.0 (0.0‐1.0)	<.001
Preoperative CEA^b^ level (log), median (IQR)	0.00 (0.0‐0.4)	1.4 (1.1‐1.9)	<.001
Postoperative chemotherapy, n (%)			<.001
FOLFOX	4631 (15.1)	308 (14.2)	
FOLFIRI	302 (1.0)	5 (0.2)	
GELOX	662 (2.1)	114 (5.3)	
Other	2138 (6.9)	1155 (53.2)	
None	22,950 (74.8)	588 (27.1)	
Postoperative radiation therapy, n (%)			<.001
No	30,153 (98.3)	2088 (96.2)	
Yes	530 (1.7)	82 (3.8)	

a *P* value was calculated using the Kolmogorov-Smirnov test for continuous variables and the chi-square test or Fisher exact test for categorical variables.

b CEA: carcinoembryonic antigen.

### Data Splitting

The synthetic data were split into source training (20,520 patients) and source validation (10,163 patients). Hospital data were split into target training (1386 patients), target validation (409 patients), and target tests (375 patients).

Synthetic data were obtained from 3 Korean institutions (161,410 records in total) and reduced to 30,683 patients after applying selection, exclusion, and preprocessing steps for model pretraining. External validation was performed using a dataset from the Hwasun Jeonnam National University Hospital, which included surgical patient data (5490 records), of which 2170 patients were included in the final analysis.

### Model Performance

The experimental results are presented in Tables 2–5. Detailed performance results for all model combinations optimized using AUPRC and *F*_1_-score are provided in Multimedia Appendix 1 and Multimedia Appendix 2, respectively. The AUPRC for models trained on synthetic data and evaluated on hospital data ranged from 0.2706 to 0.5391—domain adaptation (0.4124‐0.5391), zero-shot (0.2706‐0.4171), and ensemble (0.3919‐0.5161). In comparison, the hospital-trained baseline model showed an AUPRC of 0.3576‐0.4663. The low absolute performance of AUPRC is likely the result of a complex interplay of various factors, including moderate class imbalance, a limited feature set, and domain differences between synthetic and hospital data [36]. Therefore, we used changes relative to the baseline to evaluate the net improvement effect of external validation.

**Table 2.** Performance of colorectal cancer survival prediction models optimized for AUPRC^a^, evaluated using AUPRC and ΔAUPRC on the external hospital validation cohort.

Algorithm, sampling, and model	AUPRC	ΔAUPRC^b^ (95% CI)	*P* value^c^
LightGBM^d^			
No sampling			
Baseline	0.4025	—^e^	—
Domain adaptation	0.4336	+0.0347 (−0.0583 to +0.1346)	.48
Zero-shot	0.2706	−0.1326 (−0.2378 to −0.0299)	.02
Ensemble	0.4064	+0.0089 (−0.0636 to +0.0809)	.80
RUS^f^			
Baseline	0.3576	—	—
Domain adaptation	0.4931	+0.1334 (+0.0489 to +0.2168)	.001^g^
Zero-shot	0.3339	−0.0293 (−0.1435 to +0.0828)	.59
Ensemble	0.4605	+0.1012 (+0.0314 to +0.1778)	.004^g^
SMOTEENN^h^			
Baseline	0.4361	—	—
Domain adaptation	0.4711	+0.0298 (−0.0684 to +0.1237)	.55
Zero-shot	0.3328	−0.1094 (−0.2340 to +0.0124)	.09
Ensemble	0.4674	+0.0247 (−0.0530 to +0.1017)	.53
XGBoost^i^			
No sampling			
Baseline	0.4555	—	—
Domain adaptation	0.4781	+0.0180 (−0.0591 to +0.0994)	.66
Zero-shot	0.3597	−0.0990 (−0.2158 to +0.0179)	.097
Ensemble	0.4524	−0.0093 (−0.0763 to +0.0539)	.79
RUS			
Baseline	0.3729	—	—
Domain adaptation	0.5061	+0.1263 (+0.0387 to +0.2044)	.004^g^
Zero-shot	0.4008	+0.0222 (−0.0914 to +0.1291)	.66
Ensemble	0.5060	+0.1258 (+0.0478 to +0.2034)	.002^g^
SMOTEENN			
Baseline	0.4484	—	—
Domain adaptation	0.4295	−0.0246 (−0.1321 to +0.0810)	.64
Zero-shot	0.3896	−0.0657 (−0.1859 to +0.0550)	.27
Ensemble	0.4661	+0.0111 (−0.0875 to +0.1004)	.82

a AUPRC: area under the precision-recall curve.

b Δ values represent the mean difference estimated from 2000 bootstrap resamples of the test set, not the arithmetic difference between point estimates. 95% CIs were derived from the bootstrap distribution.

c Two-sided *P* values were derived from the bootstrap distribution.

d LightGBM: light gradient boosting machine.

e Not applicable.

f RUS: random undersampling.

g Values with improved ΔAUPRC direction and *P*<.05.

h SMOTEENN: synthetic minority oversampling technique combined with edited nearest neighbors.

i XGBoost: extreme gradient boosting.

**Table 3.** Calibration performance of colorectal cancer survival prediction models optimized for AUPRC^a^, evaluated using Brier score and ΔBrier on the external hospital validation cohort.

Model	Brier	ΔBrier^b^ (95% CI)	*P* value^c^
LightGBM^d^			
No sampling			
Baseline	0.1514	—^e^	—
Domain adaptation	0.1421	−0.0094 (−0.0217 to +0.0032)	.13
Zero-shot	0.1529	+0.0016 (−0.0151 to +0.0190)	.87
Ensemble	0.1402	−0.0112 (−0.0211 to −0.0011)	.03^f^
RUS^g^			
Baseline	0.2018	—	—
Domain adaptation	0.1977	−0.0041 (−0.0105 to +0.0024)	.21
Zero-shot	0.2287	+0.0271 (+0.0170 to +0.0368)	<.001
Ensemble	0.1887	−0.0132 (−0.0192 to −0.0068)	<.001^f^
SMOTEENN^h^			
Baseline	0.1958	—	—
Domain adaptation	0.1594	−0.0369 (−0.0625 to −0.0120)	.006^f^
Zero-shot	0.1824	−0.0132 (−0.0438 to +0.0160)	.397
Ensemble	0.1529	−0.0429 (−0.0669 to −0.0216)	<.001^f^
XGBoost^i^			
No sampling			
Baseline	0.1495	—	—
Domain adaptation	0.1588	+0.0092 (−0.0026 to +0.0206)	.13
Zero-shot	0.1841	+0.0346 (+0.0183 to +0.0506)	<.001^f^
Ensemble	0.1573	+0.0078 (−0.0014 to +0.0166)	.09
RUS			
Baseline	0.1999	—	—
Domain adaptation	0.1511	−0.0487 (−0.0611 to −0.0358)	<.001^f^
Zero-shot	0.1856	−0.0144 (−0.0272 to −0.0003)	.05^f^
Ensemble	0.1535	−0.0464 [−0.0556 to −0.0369)	<.001^f^
SMOTEENN			
Baseline	0.1930	—	—
Domain adaptation	0.1658	−0.0273 (−0.0441 to −0.0102)	.001^f^
Zero-shot	0.1675	−0.0252 (−0.0454 to −0.0042)	.02^f^
Ensemble	0.1517	−0.0415 (−0.0551 to −0.0267)	<.001^f^

a AUPRC: area under the precision-recall curve.

b Δ values represent the mean difference estimated from 2000 bootstrap resamples of the test set, not the arithmetic difference between point estimates. 95% CIs were derived from the bootstrap distribution.

c Two-sided *P* values were derived from the bootstrap distribution.

d LightGBM: light gradient boosting machine.

e Not applicable.

f Values with improved ΔBrier direction and *P*<.05.

g RUS: random undersampling.

h SMOTEENN: synthetic minority oversampling technique combined with edited nearest neighbors.

i XGBoost: extreme gradient boosting.

**Table 4.** Performance of colorectal cancer survival prediction models optimized for *F*_1_-score, evaluated using AUPRC^a^ and ΔAUPRC on the external hospital validation cohort.

Model	AUPRC	ΔAUPRC^b^ (95% CI)	*P* value^c^
LightGBM^d^			
No sampling			
Baseline	0.4339	—^e^	—
Domain adaptation	0.4124	−0.0258 (−0.1424 to +0.0899)	.66
Zero-shot	0.2765	−0.1571 (−0.2735 to −0.0395)	.006
Ensemble	0.4179	−0.0209 (−0.1223 to +0.0773)	.72
RUS^f^			
Baseline	0.3816	—	—
Domain adaptation	0.4881	+0.0996 (+0.0295 to +0.1709)	.008^g^
Zero-shot	0.3596	−0.0294 (−0.1447 to +0.0911)	.63
Ensemble	0.3919	+0.0706 (−0.0253 to +0.1575)	.15
SMOTEENN^h^			
Baseline	0.4205	—	—
Domain adaptation	0.4363	+0.0170 (−0.0785 to +0.1161)	.74
Zero-shot	0.3122	−0.1049 (−0.2131 to +0.0037)	.06
Ensemble	0.4460	−0.0299 (−0.1314 to +0.0699)	.54
XGBoost^i^			
No sampling			
Baseline	0.4642	—	—
Domain adaptation	0.4510	−0.0104 (−0.0890 to +0.0704)	.81
Zero-shot	0.3405	−0.1222 (−0.2305 to ‐0.0162)	.03
Ensemble	0.4452	−0.0193 (−0.0912 to +0.0495)	.61
RUS			
Baseline	0.3876	—	—
Domain adaptation	0.5391	+0.1474 (+0.0523 to +0.2387)	<.001^g^
Zero-shot	0.4171	+0.0290 (−0.0856 to +0.1355)	.59
Ensemble	0.5161	+0.1253 (+0.0433 to +0.2063)	.005^g^
SMOTEENN			
Baseline	0.4663	—	—
Domain adaptation	0.4524	−0.0154 (−0.1114 to +0.0772)	.74
Zero-shot	0.3763	−0.0917 (−0.2093 to +0.0200)	.12
Ensemble	0.4591	−0.0088 (−0.0983 to +0.0777)	.88

a AUPRC: area under the precision-recall curve.

b Δ values represent the mean difference estimated from 2000 bootstrap resamples of the test set, not the arithmetic difference between point estimates. 95% CIs were derived from the bootstrap distribution.

c Two-sided *P* values were derived from the bootstrap distribution.

d LightGBM: light gradient boosting machine.

e Not applicable.

f RUS: random undersampling.

g Values with improved ΔAUPRC direction and *P*<.05.

h SMOTEENN: synthetic minority oversampling technique combined with edited nearest neighbors.

i XGBoost: extreme gradient boosting.

**Table 5.** Calibration performance of colorectal cancer survival prediction models optimized for *F*_1_-score, evaluated using Brier score and ΔBrier on the external hospital validation cohort.

Algorithm, sampling, and model	Brier	ΔBrier^a^ (95% CI)	*P* value^b^
LightGBM^c^			
No sampling			
Baseline	0.1389	—^d^	—
Domain adaptation	0.1371	−0.0019 (−0.0159 to +0.0125)	.799
Zero-shot	0.1480	+0.0090 (−0.0103 to +0.0290)	.39
Ensemble	0.1433	+0.0043 (−0.0083 to +0.0160)	.49
RUS^e^			
Baseline	0.1929	—	—
Domain adaptation	0.1516	−0.0411 (−0.0577 to −0.0251)	<.001^f^
Zero-shot	0.2151	+0.0226 (−0.0032 to +0.0469)	.08
Ensemble	0.1969	+0.0041 (−0.0195 to +0.0256)	.71
SMOTEENN^g^			
Baseline	0.1951	—	—
Domain adaptation	0.1569	−0.0384 (−0.0634 to −0.0139)	<.001^f^
Zero-shot	0.1791	−0.0159 (−0.0458 to +0.0134)	.29
Ensemble	0.1710	−0.0241 (−0.0516 to +0.0008)	.06
XGBoost^h^			
No sampling			
Baseline	0.1787	—	—
Domain adaptation	0.1517	−0.0270 (−0.0394 to −0.0142)	<.001^f^
Zero-shot	0.1893	+0.0107 (−0.0020 to +0.0236)	.10
Ensemble	0.1653	−0.0134 (−0.0217 to −0.0051)	.003^f^
RUS			
Baseline	0.2080	—	—
Domain adaptation	0.1461	−0.0618 (−0.0749 to −0.0484)	<.001^f^
Zero-shot	0.1989	−0.0092 (−0.0181 to +0.0004)	.06
Ensemble	0.1680	−0.0401 (−0.0479 to −0.0320)	<.001^f^
SMOTEENN			
Baseline	0.1798	—	—
Domain adaptation	0.1566	−0.0233 (−0.0441 to −0.0023)	.03^f^
Zero-shot	0.1674	−0.0124 (−0.0388 to +0.0141)	.35
Ensemble	0.1511	−0.0287 (−0.0473 to −0.0098)	.002^f^

a Δ values represent the mean difference estimated from 2000 bootstrap resamples of the test set, not the arithmetic difference between point estimates. 95% CIs were derived from the bootstrap distribution.

b Two-sided *P* values were derived from the bootstrap distribution.

c LightGBM: light gradient boosting machine.

d Not applicable.

e RUS: random undersampling.

f Values with improved ΔBrier direction and *P*<.05.

g SMOTEENN: synthetic minority oversampling technique combined with edited nearest neighbors.

h XGBoost: extreme gradient boosting.

The domain adaptation model showed ΔAUPRC>0 in 8 out of 12 combinations, with significant improvement over the baseline model observed in 4 combinations. The largest improvement was observed in the XGBoost+RUS+*F*_1_ optimization setting (ΔAUPRC=+0.1474, 95% CI +0.0523 to +0.2387; *P*<.001; AUPRC=0.5391). For calibration performance, 11 out of 12 combinations showed ΔBrier score<0, with 8 of these demonstrating statistically significant improvement. Thus, the predicted rates of the domain adaptation model matched the actual occurrence rates, even in the external hospital distribution.

The ensemble model, serving as a comparator group, showed ΔAUPRC>0 in 7 out of 12 combinations, with statistically significant improvement over the baseline model observed in 3 combinations. The largest improvement was observed in the XGBoost+RUS+AUPRC optimization (ΔAUPRC=+0.1258, 95% CI +0.0478 to +0.2034; *P=*.002; AUPRC=0.5060). Calibration performance showed ΔBrier score<0 in 9 out of 12 combinations, with 8 being statistically significant, demonstrating results similar to those of the domain adaptation model.

In contrast, the zero-shot model consistently showed poor discrimination performance. Only 2 of 12 combinations exhibited ΔAUPRC>0, and none of these improvements were statistically significant. The remaining 10 combinations showed no improvement, including 3 with statistically significant degradation. For the ΔBrier score, 6 out of 12 combinations showed performance decline, with 2 combinations being significant.

Although absolute performance was constrained by the limited number of shared features, certain model combinations significantly outperformed the baseline. The performance of the zero-shot model was limited and inconsistent, and there was no statistically significant improvement. In some combinations, performance actually dropped significantly. In contrast, models that directly applied domain adaptation (domain adaptation models) or models using domain adaptation (ensemble models) showed performance improvements across various combinations. Statistically significant performance improvements were observed in some settings. This confirms the clinical value of synthetic data when the distribution of target data is leveraged.

## Discussion

### Principal Findings

This study evaluated the feasibility and limitations of survival prediction models using specific synthetic data (NCDC synthetic dataset) in a privacy-constrained environment. After pretraining the model using large-scale synthetic data, we applied domain adaptation using single-center clinical data. The results showed that domain adaptation using real clinical data can partially compensate for the performance observed in the synthetic data-based zero-shot model used in this study. This suggests that synthetic data alone cannot replace real clinical data in the setting of this study, but when combined with limited real data, it can be used as a supplementary resource to improve model transfer performance.

### Comparison With Previous Work

Previous studies have mainly focused on domain adaptation between multicenter real-world clinical data, and relatively few studies have evaluated the transfer performance to real-world clinical data after using synthetic data as the main training resource. This study differs from previous studies in that we used generated and publicly available NCDC synthetic data for pretraining and applied a strategy to calibrate it with single-institution data. As reported in previous studies, synthetic data may not sufficiently reproduce the distribution of rare or clinically important variables depending on how they are generated. We show empirically in an external validation setting that this limitation can lead to distributional differences between synthetic data and real-world hospital data, which can manifest as poor zero-shot model performance. This demonstrates the potential of synthetic data-based pretraining learning strategies, as well as the limitations due to data quality and domain differences, with concrete examples.

### Limitations

This study had several limitations. First, there are limitations regarding the quality and distributional representativeness of the synthetic data used in this study. Significant discrepancies were observed in the prevalence of perineural invasion (PNI) between the NCDC synthetic data and hospital data. PNI is a well-established prognostic factor in predicting cancer survival. In addition, the synthetic dataset used in this study is a publicly available dataset, and a significant number of records were excluded from the analysis due to missing essential covariates. The NCDC synthetic data is RTSGAN-based and publicly available, and the distributional skew and missingness issues are consistent with the nature of RTSGAN-based synthetic data, where the goal is to preserve global statistical distribution. Previous studies have repeatedly reported underrepresentation of rare or clinically important variables as a limitation of synthetic data generation models [37]. It has also been reported that missingness in synthetic data may not be a newly generated problem, but rather a reflection of missingness mechanisms in the original clinical data or structural limitations of the generation model [38,39]. In this study, we did not artificially correct for these distributional skewnesses and missingnesses but rather performed the analysis while reflecting the realistic characteristics of the available synthetic data. Distributional differences in key prognostic factors and sample loss due to missingness may have partially contributed to the poorer predictive performance observed in the zero-shot transition setting. Additionally, differences in the distribution of major clinical variables were also observed between the synthetic dataset and the hospital dataset. In particular, there was a significant difference in the distribution of histological subtypes. While 97.2% of the hospital cohort was classified as adenocarcinoma, only 55.6% of the synthetic cohort was classified as adenocarcinoma, with the remainder consisting of nonadenocarcinoma subtypes, such as mucinous carcinoma and carcinoid tumors. Differences were also observed in postoperative chemotherapy patterns. These included regimen changes, combination or sequential therapies, and institution-specific treatment patterns not predefined, reflecting heterogeneous real-world treatment practices. These inconsistencies may have contributed to the observed performance differences and limitations in transferability between datasets. Second, the results of this study do not represent a direct performance comparison with an optimized clinical prediction model that uses all clinical variables included in the hospital data. The hospital data–based baseline model used in this study is a feature-constrained baseline model for comparison that is limited to 12 common variables to maintain commonality with synthetic data. Therefore, the performance improvement of domain adaptation models observed in this study should be interpreted as a validation of the methodological efficiency of synthetic data-based pretraining learning and model transfer strategies under the same input constraints, not as a claim of clinical superiority over local models that use all clinical information. Third, the external validation of this study was conducted using data from a single health care organization, which does not sufficiently validate its applicability to a variety of health care settings. Multicenter validation is needed to confirm the transferability and domain adaptation of synthetic data in settings with different institutional sizes, geographic characteristics, and patient populations. In addition, while the approximately 80:20 survival-to-death ratio observed for 7-year survival prediction is not an extreme level of class imbalance, the limited number of variables and domain differences between synthetic and hospital data, combined with the single-institution validation environment, may have formed an upper bound on model performance. Considering these characteristics, we chose AUPRC as the main evaluation metric to more appropriately reflect the discriminative performance of the model in an unbalanced clinical event prediction environment, while simultaneously using the Brier score to assess the degree of calibration of the prediction probability, which is important in clinical risk prediction. However, despite this choice of metrics, the absolute performance of the models remained in a limited range. In fact, the absolute performance of the model based on synthetic data ranged from an AUPRC of 0.3070 to 0.5165 and an *F*_1_-score of 0.3391 to 0.5132, while the baseline model using only hospital data had a similarly wide performance range with an AUPRC of 0.3975 to 0.458 and an *F*_1_-score of 0.4231 to 0.4751. This suggests that the performance limitations observed in this study may be the result of a combination of structural constraints of single-site validation, variable limitations, and domain differences, rather than simple class imbalance. Fourth, while age can be used as a continuous variable, we binned it in this study to increase distributional stability and mitigate nonlinear effects between synthetic and hospital data. This approach was intended to simplify interactions between variables in the tree-based model and reduce sensitivity to distributional differences between domains. However, this can be interpreted as a limitation of this study in that some continuous information may be lost.

### Future Work

Future work should extend analyses using public synthetic data, such as this study, to more systematically evaluate the applicability and limitations of synthetic data-based pretraining learning and model transfer strategies using real-world clinical data. In particular, the impact of distributional mismatches or missing characteristics of clinically important rare variables on model performance should be quantitatively analyzed, and data usage and transfer strategies that can mitigate these issues should be further explored. Furthermore, by introducing a quality assessment framework that considers how the missingness mechanisms of the original clinical data are reflected in the synthetic data, it is expected that the information loss that may occur when using synthetic data can be interpreted more clearly. Furthermore, external validation using multicenter real-world data will validate the generalizability of the synthetic data-based transfer learning approach presented in this study, and studies extending it to other diseases and prediction tasks will contribute to a broader examination of the conditional use of synthetic data.

### Conclusion

In this study, we define NCDC synthetic data not as a stand-alone learning resource, but as an auxiliary resource for pretraining learning that can be used in a privacy-preserving environment and demonstrate that it can improve prediction performance when combined with real-world clinical data through domain adaptation. In the setting of this study, the results clearly highlight the limitations of synthetic data-based zero-shot models, while confirming that limited real-world data plays a key role in model transfer. These results suggest that synthetic data are not a substitute for independent clinical application but can be used as a complement to increase methodological efficiency when combined with real-world clinical data with limited sample size and variable composition. This supports that the purpose of this study was not to replace real-world clinical decision-making, but to evaluate the methodological feasibility of synthetic data-based transfer learning strategies. Recent studies have emphasized that reproducibility and computational efficiency are also important quality criteria for medical AI research involving large-scale synthetic data and iterative model evaluation [35]. From this perspective, the fixed software environment and systematic experimental pipeline design of this study can be interpreted as a foundation for ensuring the sustainability and reproducibility of future synthetic data-driven clinical AI research.

## Supplementary material

10.2196/86087 Multimedia Appendix 1 Detailed performance results of colorectal cancer survival prediction models under AUPRC-based optimization across algorithms, sampling strategies, and model types.

10.2196/86087 Multimedia Appendix 2 Detailed performance results of colorectal cancer survival prediction models under *F*_1_-score–based optimization across algorithms, sampling strategies, and model types.

## Abbreviations

AUPRC: area under the precision-recall curve
LightGBM: light gradient boosting machine
NCDC: National Cancer Data Center
PNI: perineural invasion
RTSGAN: real-world time-series generative adversarial network
RUS: random undersampling
SMOTEENN: synthetic minority oversampling technique combined with edited nearest neighbors
XGBoost: extreme gradient boosting
