# Correction: Evaluation of the Applicability of Synthetic Data in the Development of Colorectal Cancer Survival Prediction Models: External Validation of Advanced Machine Learning Models Based on National Cancer Data Center Data

**DOI:** 10.2196/110851

**Published:** 2026-10-30

**Authors:** Yujeong Jang, Jae Hoon Kwon, Heeyong Kim, You-Jin Joung, Junho ‍Nang, Chang Hyun Kim

**Affiliations:** 1 Department of Regulatory Science, Kyung Hee University, Seoul, Republic of Korea; 2 Department of Surgery, Chonnam National University Hwasun Hospital, 322 Seoyang-ro, Hwasun, 58128, Republic of Korea; 3 C&R Research, Seoul, Republic of Korea; 4 Clinical Trial Center, Institute for Biomedical Science, Chonnam National University Hwasun Hospital, 322, Seoyang-ro, Hwasun-eup, Hwasun-gun, Jeollanam-do, Republic of Korea, 82 10-8940-1365

In “Evaluation of the Applicability of Synthetic Data in the Development of Colorectal Cancer Survival Prediction Models: External Validation of Advanced Machine Learning Models Based on National Cancer Data Center Data” [1], the authors noted one error.

The Funding section was revised from the following:

*This research was supported by the Korea-Hwasun Oncology Precision Biomedicine & Experimental Trials Project, a research and development project commissioned by the Ministry of Health and Welfare of the Republic of Korea (grant RS-2025-00433283), and by the Development of an AI-Driven Platform for Clinical Validation in Senior Healthcare Focused on Degenerative Brain Diseases through the Korea Planning and Evaluation Institute of Industrial Technology (grant RS-2024-00433283).*

The corrected Funding section now reads:

*This research was supported by the SmartTech Clinical Research Center under the Korea Health Technology R&D Project through the Korea Health Industry Development Institute (KHIDI), funded by the Ministry of Health & Welfare, Republic of Korea (grant RS-2025‐19252970), and by the Development of an AI-Driven Platform for Clinical Validation in Senior Healthcare Focused on Degenerative Brain Diseases through the Korea Planning and Evaluation Institute of Industrial Technology (grant RS-2024‐00433283).*

The correction will appear in the online version of the paper on the JMIR Publications website together with the publication of this correction notice. Because this was made after submission to PubMed, PubMed Central, and other full-text repositories, the corrected article has also been resubmitted to those repositories.
