# Supplementary material for: Evaluation of the Applicability of Synthetic Data in the Development of Colorectal Cancer Survival Prediction Models: External Validation of Advanced Machine Learning Models Based on National Cancer Data Center Data
Source: J Med Internet Res. 2026 Jul 7;28:e86087. doi: 10.2196/86087 (PMC13340573; doi:10.2196/86087)
Supplement: Multimedia Appendix 1 [file jmir-v28-e86087-s001.docx]

**Multimedia Appendix 1. Detailed performance results of colorectal cancer survival prediction models under AUPRC-based optimization across algorithms, sampling strategies, and model types**

| **Algorithm** | **Sampling** | **Performance Metrics** | **Baseline** | **Domain adaptation** | **Zero-shot** | **Ensemble** |
| --- | --- | --- | --- | --- | --- | --- |
| **LightGBM** | **No sampling** | **AUROC^a^** | 0.7233 | 0.7419 | 0.6404 | 0.7392 |
|  |  | **Precision** | 0.3824 | 0.3191 | 0.2642 | 0.3942 |
|  |  | **Recall** | 0.3714 | 0.6429 | 0.6000 | 0.5857 |
|  |  | **F1-score** | 0.3768 | 0.4265 | 0.3668 | 0.4713 |
|  |  | **F2-score** | 0.5705 | 0.5378 | 0.5373 | 0.5605 |
|  |  | **Accuracy** | 0.7713 | 0.6782 | 0.6144 | 0.7553 |
|  |  | **MCC^b^** | 0.2368 | 0.2646 | 0.1715 | 0.3305 |
|  |  | **Specificity** | 0.8627 | 0.6863 | 0.6176 | 0.7941 |
|  | **RUS** | **AUROC^a^** | 0.7144 | 0.6689 | 0.7524 | 0.7569 |
|  |  | **Precision** | 0.3548 | 0.2560 | 0.3939 | 0.3566 |
|  |  | **Recall** | 0.4714 | 0.6143 | 0.5571 | 0.6571 |
|  |  | **F1-score** | 0.4049 | 0.3613 | 0.4615 | 0.4623 |
|  |  | **F2-score** | 0.5816 | 0.4799 | 0.5703 | 0.5603 |
|  |  | **Accuracy** | 0.7420 | 0.5957 | 0.7580 | 0.7154 |
|  |  | **MCC^b^** | 0.2484 | 0.1611 | 0.3191 | 0.3164 |
|  |  | **Specificity** | 0.8039 | 0.5915 | 0.8039 | 0.7288 |
|  | **SMOTEENN** | **AUROC^a^** | 0.7434 | 0.7484 | 0.6680 | 0.7441 |
|  |  | **Precision** | 0.4211 | 0.2793 | 0.2525 | 0.2907 |
|  |  | **Recall** | 0.5714 | 0.7143 | 0.7286 | 0.7143 |
|  |  | **F1-score** | 0.4848 | 0.4016 | 0.3750 | 0.4132 |
|  |  | **F2-score** | 0.5731 | 0.5790 | 0.5461 | 0.5611 |
|  |  | **Accuracy** | 0.7739 | 0.6037 | 0.5479 | 0.6223 |
|  |  | **MCC^b^** | 0.3509 | 0.2281 | 0.1835 | 0.2466 |
|  |  | **Specificity** | 0.8203 | 0.5784 | 0.5065 | 0.6013 |
| **XGBoost** | **No sampling** | **AUROC^a^** | 0.7679 | 0.7677 | 0.6726 | 0.7628 |
|  |  | **Precision** | 0.4337 | 0.4235 | 0.2488 | 0.3793 |
|  |  | **Recall** | 0.5143 | 0.5143 | 0.7571 | 0.6286 |
|  |  | **F1-score** | 0.4706 | 0.4645 | 0.3746 | 0.4731 |
|  |  | **F2-score** | 0.5773 | 0.5901 | 0.5446 | 0.5938 |
|  |  | **Accuracy** | 0.7846 | 0.7793 | 0.5293 | 0.7394 |
|  |  | **MCC^b^** | 0.3385 | 0.3296 | 0.1840 | 0.3314 |
|  |  | **Specificity** | 0.8464 | 0.8399 | 0.4771 | 0.7647 |
|  | **RUS** | **AUROC^a^** | 0.7288 | 0.7788 | 0.7038 | 0.7732 |
|  |  | **Precision** | 0.3864 | 0.4634 | 0.2698 | 0.3701 |
|  |  | **Recall** | 0.4857 | 0.5429 | 0.7286 | 0.6714 |
|  |  | **F1-score** | 0.4304 | 0.5000 | 0.3938 | 0.4772 |
|  |  | **F2-score** | 0.5755 | 0.6067 | 0.5375 | 0.5882 |
|  |  | **Accuracy** | 0.7606 | 0.7979 | 0.5824 | 0.7261 |
|  |  | **MCC^b^** | 0.2843 | 0.3762 | 0.2161 | 0.3374 |
|  |  | **Specificity** | 0.8235 | 0.8562 | 0.5490 | 0.7386 |
|  | **SMOTEENN** | **AUROC^a^** | 0.7577 | 0.7353 | 0.6924 | 0.7550 |
|  |  | **Precision** | 0.3942 | 0.3545 | 0.2660 | 0.3630 |
|  |  | **Recall** | 0.5857 | 0.5571 | 0.7714 | 0.7000 |
|  |  | **F1-score** | 0.4713 | 0.4333 | 0.3956 | 0.4780 |
|  |  | **F2-score** | 0.5675 | 0.5743 | 0.5567 | 0.5894 |
|  |  | **Accuracy** | 0.7553 | 0.7287 | 0.5612 | 0.7154 |
|  |  | **MCC^b^** | 0.3305 | 0.2782 | 0.2222 | 0.3399 |
|  |  | **Specificity** | 0.7941 | 0.7680 | 0.5131 | 0.7190 |

^a^AUROC: area under the receiver operating characteristic curve.

^b^MCC: Matthews correlation coefficient.
