# Supplementary material for: Evaluation of the Applicability of Synthetic Data in the Development of Colorectal Cancer Survival Prediction Models: External Validation of Advanced Machine Learning Models Based on National Cancer Data Center Data
Source: J Med Internet Res. 2026 Jul 7;28:e86087. doi: 10.2196/86087 (PMC13340573; doi:10.2196/86087)
Supplement: Multimedia Appendix 2 [file jmir-v28-e86087-s002.docx]

**Multimedia Appendix 2. Detailed performance results of colorectal cancer survival prediction models under F1-score–based optimization across algorithms, sampling strategies, and model types**

| **Algorithm** | **Sampling** | **Performance Metrics** | **Baseline** | **Domain adaptation** | **Zero-shot** | **Ensemble** |
| --- | --- | --- | --- | --- | --- | --- |
| **LightGBM** | **No sampling** | **AUROC^a^** | 0.7488 | 0.7026 | 0.6121 | 0.7080 |
|  |  | **Precision** | 0.4186 | 0.3187 | 0.2465 | 0.3444 |
|  |  | **Recall** | 0.5143 | 0.4143 | 0.5000 | 0.4429 |
|  |  | **F1-score** | 0.4615 | 0.3602 | 0.3302 | 0.3875 |
|  |  | **F2-score** | 0.5796 | 0.5134 | 0.4912 | 0.5475 |
|  |  | **Accuracy** | 0.7766 | 0.7261 | 0.6223 | 0.7394 |
|  |  | **MCC^b^** | 0.3252 | 0.1924 | 0.1207 | 0.2281 |
|  |  | **Specificity** | 0.8366 | 0.7974 | 0.6503 | 0.8072 |
|  | **RUS** | **AUROC^a^** | 0.7230 | 0.7494 | 0.6561 | 0.7240 |
|  |  | **Precision** | 0.3699 | 0.4762 | 0.2626 | 0.3162 |
|  |  | **Recall** | 0.3857 | 0.4286 | 0.3775 | 0.6143 |
|  |  | **F1-score** | 0.3776 | 0.4511 | 0.5212 | 0.4175 |
|  |  | **F2-score** | 0.5882 | 0.5531 | 0.6714 | 0.5653 |
|  |  | **Accuracy** | 0.7633 | 0.8059 | 0.5878 | 0.6809 |
|  |  | **MCC^b^** | 0.2316 | 0.3343 | 0.1871 | 0.2514 |
|  |  | **Specificity** | 0.8497 | 0.8922 | 0.5686 | 0.6961 |
|  | **SMOTEENN** | **AUROC** | 0.7232 | 0.7499 | 0.6793 | 0.7280 |
|  |  | **Precision** | 0.4328 | 0.4430 | 0.3056 | 0.3778 |
|  |  | **Recall** | 0.4143 | 0.5000 | 0.6286 | 0.4857 |
|  |  | **F1-score** | 0.4234 | 0.4698 | 0.4112 | 0.4250 |
|  |  | **F2-score** | 0.5689 | 0.5743 | 0.5449 | 0.5567 |
|  |  | **Accuracy** | 0.7899 | 0.7899 | 0.6649 | 0.7553 |
|  |  | **MCC^b^** | 0.2951 | 0.3403 | 0.2416 | 0.2761 |
|  |  | **Specificity** | 0.8758 | 0.8562 | 0.6732 | 0.8170 |
| **XGBoost** | **No sampling** | **AUROC^a^** | 0.7579 | 0.7701 | 0.6881 | 0.7612 |
|  |  | **Precision** | 0.4318 | 0.4359 | 0.2812 | 0.3622 |
|  |  | **Recall** | 0.5429 | 0.4857 | 0.5143 | 0.3622 |
|  |  | **F1-score** | 0.4810 | 0.4595 | 0.3636 | 0.4670 |
|  |  | **F2-score** | 0.5808 | 0.6200 | 0.5577 | 0.5852 |
|  |  | **Accuracy** | 0.7819 | 0.7872 | 0.6649 | 0.7207 |
|  |  | **MCC^b^** | 0.3488 | 0.3282 | 0.1755 | 0.3230 |
|  |  | **Specificity** | 0.8366 | 0.8562 | 0.6993 | 0.7353 |
|  | **RUS** | **AUROC^a^** | 0.7455 | 0.7967 | 0.7275 | 0.7814 |
|  |  | **Precision** | 0.3763 | 0.4483 | 0.2919 | 0.4255 |
|  |  | **Recall** | 0.5000 | 0.5571 | 0.6714 | 0.5714 |
|  |  | **F1-score** | 0.4294 | 0.4968 | 0.4069 | 0.4878 |
|  |  | **F2-score** | 0.5888 | 0.5989 | 0.5792 | 0.5832 |
|  |  | **Accuracy** | 0.7527 | 0.7899 | 0.6356 | 0.7766 |
|  |  | **MCC^b^** | 0.2801 | 0.3695 | 0.2351 | 0.3550 |
|  |  | **Specificity** | 0.8105 | 0.8431 | 0.6275 | 0.8235 |
|  | **SMOTEENN** | **AUROC^a^** | 0.7652 | 0.7628 | 0.7005 | 0.7567 |
|  |  | **Precision** | 0.3978 | 0.5588 | 0.3012 | 0.3704 |
|  |  | **Recall** | 0.5286 | 0.2714 | 0.7143 | 0.7143 |
|  |  | **F1-score** | 0.4540 | 0.3654 | 0.4237 | 0.4878 |
|  |  | **F2-score** | 0.6063 | 0.5955 | 0.5488 | 0.5980 |
|  |  | **Accuracy** | 0.7633 | 0.8245 | 0.6383 | 0.7207 |
|  |  | **MCC^b^** | 0.3117 | 0.3019 | 0.2628 | 0.3542 |
|  |  | **Specificity** | 0.8170 | 0.9510 | 0.6209 | 0.7222 |

^a^AUROC: area under the receiver operating characteristic curve.

^b^MCC: Matthews correlation coefficient.
